# Extraction and Physicochemical Characterization of Dried Powder Mucilage from *Opuntia ficus-indica* Cladodes and Aloe Vera Leaves: A Comparative Study

**DOI:** 10.3390/polym13111689

**Published:** 2021-05-22

**Authors:** María Carolina Otálora, Andrea Wilches-Torres, Jovanny A. Gómez Castaño

**Affiliations:** 1 Grupo de Investigación en Ciencias Básicas (NÚCLEO), Facultad de Ciencias e Ingeniería, Universidad de Boyacá, Tunja, Boyacá 050030, Colombia; andreawilches@uniboyaca.edu.co; 2 Grupo Química-Física Molecular y Modelamiento Computacional (QUIMOL^®^), Facultad de Ciencias, Universidad Pedagógica y Tecnológica de Colombia (UPTC), Avenida Central del Norte, Tunja, Boyacá 050030, Colombia

**Keywords:** mucilage, pectin polysaccharide, *Opuntia ficus-indica*, aloe vera, acemannan, Cactaceae, Asphodelaceae

## Abstract

Cactaceae and Asphodelaceae are native desert plants known for their high mucilage content, which is a polysaccharide of growing interest in the food, cosmetic, and pharmaceutical industries. In this study, powdered mucilage was obtained from cladodes of *Opuntia ficus-indica* (OFI) and aloe vera (AV) leaves, and their molecular, morphological, and thermal properties were investigated and compared. Additionally, their dietary fiber content was determined. Three-dimensional molecular models were calculated for both mucilages using ab initio methods. Vibrational spectra (FTIR and Raman) revealed intramolecular interactions and functional groups that were specified with the help of theoretical ab initio and semi-empirical calculations. SEM micrographs measured at magnifications of 500× and 2000× demonstrated significantly different superficial and internal morphologies between these two mucilages. Thermal analysis using DSC/TGA demonstrated superior thermal stability for the OFI mucilage. The dietary fiber content in OFI mucilage was more than double that of AV mucilage. Our results show that both dehydrated mucilages present adequate thermal and nutritional properties to be used as functional ingredients in industrial formulations; however, OFI mucilage exhibited better physicochemical and functional characteristics than AV mucilage as a raw material.

## 1. Introduction

Mucilages are complex polymeric substances that are carbohydrates in nature and have garnered considerable interest in the food, pharmaceutical, and cosmetic industries for their functional, health, and nutritional benefits. Their composition is mainly based on branched polysaccharide macromolecules that form intricate molecular networks capable of retaining large amounts of water, making them a potential source of natural hydrocolloids that provide a substantial thickening effect that is desirable for the chemical and cosmetic industries [1,2]. They have medicinal attributes such us wound, burn, and ulcer healing, as well as antidiabetic and antiglycation effects [3,4]. Other mucilage applications include its use in foods as a stabilizer, flavoring agent, fat substitute [5], and edible coating to extend the useful life of fruit [6], among others [7,8].

For centuries the plants belonging to the Cactaceae and Asphodelaceae botanical families have been recognized for their abundant content of mucilage (commonly called gel) [9]. Among the most representative members of these families are the nopal cactus (*Opuntia ficus-indica* (L.) Mill) and the aloe vera plant (*Aloe barbadensis* Miller), the mucilages of which are widely renowned because of their varied nutritional, cosmetic, and medicinal properties. Mucilage obtained from *O. ficus-indica* (OFI mucilage) has been reported to be able to lower cholesterol levels [10,11] and exhibit hypoglycemic effects [12] as well as antiulcer [13], hepatoprotective, antigenotoxic, and cardioprotective pharmacological effects [14]. OFI mucilage is also considered a potent antioxidant due to its carbohydrate and polyphenol content [15,16]. Additionally, a high total dietary fiber content, between 57 and 72 g/100 g dry matter, has been reported for OFI mucilage [17,18,19].

On the other hand, numerous biological activities, including antiviral, antibacterial, laxative, radiation protection, wound healing, antioxidant, anti-inflammatory, anticancer, antidiabetic, antiallergic, and immunostimulatory effects, among others, have been attributed to the aloe vera mucilage (AV mucilage) [20,21,22,23].

The mucilage extracted from the cladodes of *Opuntia ficus-indica* is a heteropolysaccharide of an anionic polyelectrolyte nature, with a molar mass of 3 × 10^6^ g/mol, containing L-arabinose (24.6–42.0%), D-galactose (21.0–40.1%), D-xylose (22.0–22.2%), L-rhamnose (7.0–13.1%), and α-D-(1 → 4) galacturonic acid (8.0–12.7%), as well as insoluble (lignin and polysaccharides) and soluble (neutral sugars and uronic acid) fiber [5]. This natural hydrocolloid has garnered growing industrial interest due to its edible, biodegradable, non-toxic characteristics, and its cost effectiveness [24]. Its potential applications in the food, pharmaceutical, and cosmetic industries include its use as thickening, binding, emulsifying, stabilizing, and gelling agent [25]; film-former [26]; and as wall material for encapsulation processes [18,19,27]. Previous studies have shown that OFI mucilage is considered a rich source in polyunsaturated fatty acid, especially α-linolenic (C18: 3 ω-3) and linoleic (C18: 2 ω-6), which is essential for human nutrition [16].

On the other hand, the mucilage extracted from the hydroparenchyma of the succulent leaves of aloe vera is a biopolymer mainly constituted by acemannan and pectic substances [28]. The acemannan polysaccharide, with a molecular weight of around 40–50 kDa, contains partially acetylated mannose units (>60%), glucose (∼20%), and galactose (<10%) [29,30]. Previous studies have shown that the AV mucilage is considered a rich source in dietary fiber (35.5%) [28] as well as a potent antioxidant due to its aloe-emodin content [31]. This hydrocolloid also has potential use in the food industry as an edible coating [32,33,34], biodegradable film [35], and encapsulating agent [36].

Most of the reports on OFI and AV mucilages have focused on the biological properties and possible applications of these biopolymers in their fresh form or in hydrated preparations, while much less is known about their chemical and physical characteristics, as well as the possible uses of their concentrated powder. This study focuses on comparatively evaluating the structural, morphological, and thermal properties, as well as the total dietary fiber content of hot air-dried mucilages extracted from OFI cladodes and AV leaves. These two mucilages have been particularly selected for the present study given their potential usefulness as functional ingredients in formulations for nutritional, health, medicinal, and cosmetic purposes [37], as well as the scalability of the cultivation of OFI and AV species in arid and semi-arid areas [1,2]. The results of this study seek to contribute to the determination of the structural and thermal behavior of these powdered mucilages, which constitute a fundamental factor in guaranteeing their chemical stability within a food, cosmetic, or pharmacological matrix until their consumption or final application.

## 2. Materials and Methods

### 2.1. Vegetal Materials

Cladodes of *Opuntia ficus-indica* were collected in the rural jurisdiction of the city of Duitama in the Department of Boyacá, Colombia. Aloe vera leaves were purchased at a local supermarket in the city of Tunja in the Department of Boyacá, Colombia. The size of the leaves ranged between 30 and 40 cm in length.

### 2.2. Mucilage Extraction

For the extraction of mucilage from cladodes of *Opuntia ficus-indica*, the methodology reported by Quinzio et al. [38] was used with some modifications, as explained below. The cladodes were washed with distilled water, transversally cut with a Teflon knife, and manually peeled. The cladode outer layer was removed, leaving only the white and inner part (i.e., the medulla). The medulla was cut into small pieces and placed in a 1000 mL beaker. Distilled water at room temperature was added to the beaker in a 1:2 *v/v* (medulla:water) ratio, and the mixture was left for 24 h. According to the literature [1,39,40,41], these extraction parameters ensure maximum viscosity and high yield of the extracted mucilage. The hydrated medulla pieces were manually squeezed through a nylon cloth. The obtained gel was centrifuged at 10,000 rpm for 30 min, and then 95% ethanol (provided by Merck, Darmstadt, Germany) was added in a ratio of 3:1 (ethanol/centrifuged gel) at room temperature for the precipitation of the mucilage. Extraction with ethanol was used to dissolve the chlorophyll present in the pads and produce a white mucilage powder, that is, with a presentation similar to the other gums used in the food industry [5]. The precipitate was dried in Petri dishes at 105 °C in an oven (UM 400, Memmert, Schwabach, Germany) for 24 h. The dried material was manually macerated in a porcelain mortar until a fine powder was obtained. The OFI powdered mucilage was placed in high-density polyethylene bags and stored in a desiccator at room temperature with a relative humidity of 30% until use.

The aloe vera leaves were washed with distilled water at room temperature, and then the epidermis was carefully separated from the inner pulp using a Teflon knife. The pulp filets were cut into small pieces and manually squeezed to extract the leaf gel. The gel obtained was filtered through a nylon-cloth. Then 95% ethanol was added at room temperature to the filtered gel in an ethanol:gel ratio of 3:1 *v/v*, and then the mixture was stirred manually with a glass rod until the appearance of a white-milky gel (mucilage) was present. The mucilage gel was placed in Petri dishes and dried at 60 °C in an oven (UM 400, Memmert, Schwabach, Germany) for 24 h. According to literature [42], at a drying temperature of 60–70 °C a high quality aloe vera gel is obtained, that is, with minor alterations in its physicochemical and nutritional properties. The dried material was manually macerated in a porcelain mortar until a fine powder was obtained. The AV powdered mucilage was placed in high-density polyethylene bags and stored in a desiccator at room temperature with a relative humidity of 30% until use.

### 2.3. Structural Characterization

#### 2.3.1. Fourier-Transform Infrared (FTIR) Spectroscopy

The infrared spectra of the powdered mucilages of *Opuntia ficus-indica* and aloe vera were recorded on a Shimadzu Prestigie 21 spectrophotometer (Duisburg, Germany) equipped with a Michelson-type interferometer, a KBr/Ge beam-splitter, a ceramic lamp, and a DLATGS detector. The FTIR spectra were measured in the range of 4500–4520/cm with a resolution of 3.0/cm and 30 cumulative scans using the attenuated total reflectance/reflection (ATR) technique.

#### 2.3.2. Raman Spectroscopy

The surface composition of the *Opuntia ficus-indica* and aloe vera powdered mucilages were analyzed using a Raman spectrophotometer (DXR™ Smart Raman, Thermo Scientific, Waltham, MA, USA) equipped with a 785 nm excitation diode laser. Spectra were measured with an average scan time of 1.0 s using a laser power of 20.0 mW. A total of 20 scans per spectra was performed in order to improve the signal-to-noise ratio.

#### 2.3.3. Scanning Electron Microscopy (SEM)

The microscopic morphology of the powdered mucilages of *Opuntia ficus-indica* and aloe vera was evaluated by scanning electron microscopy (SEM) using EVO MA 10-Carl Zeiss equipment (Oberkochen, Germany) operating at 20 kV. All samples were coated by gold–palladium sputtering before their examination.

### 2.4. Thermal Properties

Thermogravimetric analysis (TGA)/differential scanning calorimetry (DSC) was performed on a TA Instrument (SDT Q600 V20.9 Build 20, New Castle, DE, USA). Argon was used as a purge gas (100 mL/min). The dried samples of OFI and AV mucilages were placed in aluminum pans and heated from 20 to 600 °C at a heating rate of 10 °C/min.

### 2.5. Dietary Fiber Content

Total dietary fiber (TDF) content in *Opuntia ficus-indica* and aloe vera powdered mucilage samples was determined using a total dietary fiber test kit (TDF-100A), provided by Sigma Aldrich (St. Louis, MO, USA), which is based on the enzymatic–gravimetric method AOAC 985.29 [43]. The dry, fat-free mucilage powder was gelatinized with the thermostable α-amylase and then enzymatically digested with protease and amyloglucosidase to remove protein and starch. The soluble fiber was precipitated with ethanol; then the residues were washed with ethanol and acetone, filtered, dried, and weighed. The dry material was determined for protein and ash content. Total dietary fiber (TDF) was calculated as the residue minus the weight of protein and ash and was expressed as g/100 g of powder.

### 2.6. Molecular Models and Quantum Chemical Calculations

The 3D molecular models of the mucilages were initially built in the Chem3D Pro 12.0 program [44] taking into consideration the three-dimensional conformations of the monosaccharide structures as reported in the PubChem database of the National Center for Biotechnology Information (NCBI) [45] and their preliminary minimized structures using the force field calculation method MM2 [46] as implemented in Chem3D Pro 12.0.

All quantum chemical calculations were performed using the Gaussian 09 software package (Rev. A.02) [47], while molecular models and simulated IR and Raman spectra were visualized through the GaussView 6.0.16 graphical user interface [48]. The minima structures and vibrational harmonic frequencies for acemannan and OFI mucilage polysaccharides were calculated in the gas phase using the HF/6-31(d) and the HF/6-31(d)//PM6 approximation levels of theory, respectively, assuming a temperature of 298.15 K and 1 atmosphere of pressure. The equilibrium structures were confirmed as minima in the potential energy surface by the absence of imaginary frequencies.

## 3. Results and Discussion

### 3.1. Molecular Modelling of the Isolated Dehydrated OFI and AV Mucilages

**Figure 1:**
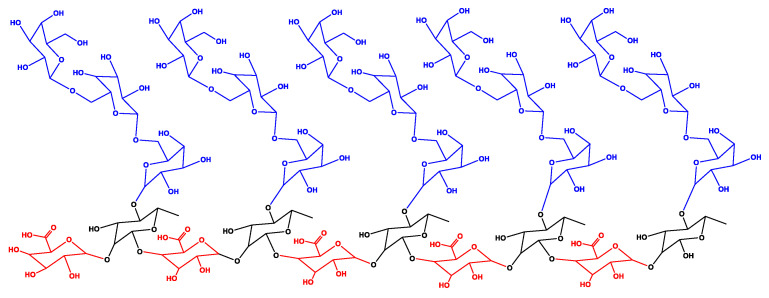
Two-dimensional representation used for the construction of the molecular model of the isolated OFI mucilage. Gal-β (in blue), Rha-β (in black), and Gal-Ac-α (in red).

To achieve a representative isolated molecular model for the OFI mucilage, a branched biopolymeric structure was built and made up of a total of 25 sugar residues that were linked according to the type and arrangement of the glycosidic bonds reported for this heteropolysaccharide [49]. The sugar residues used were β-D-galactopyranose (Gal-β), β-L-rhamnopyranose (Rha-β), and α-D-galactopyranosyl uronic acid (Gal-Ac-α) in a proportion of 60, 20, and 20%, respectively. The central chain consisted of 10 residues with intercalated Gal-Ac-α(1→2)Rha-β(1→4) glycosidic bonds, while five branched chains, each one made up of Gal-β(1→6)Gal-β(1→6)Gal-β glycosidic bonds, were connected through Gal-β(1→4)Rha-β bonds to the central chain. A two-dimensional representation of the molecular model for the OFI mucilage is presented in Figure 1.

**Figure 2:**
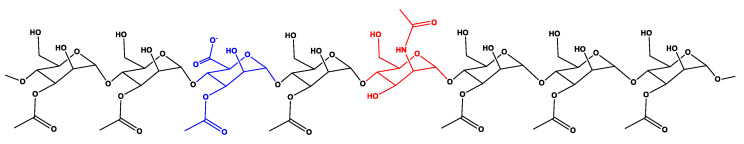
Two-dimensional representation used for the construction of the molecular model of the isolated acemannan (carrisyn). Sugar residues: α-D-galacto mannan (in black), α-D-galacto mannan acetate (in blue), and N-acetyl-D-glucosamine (in red).

To approximate the molecular model for the dry AV mucilage, an isolated structure of acemannan was built considering the type and disposition of the glycosidic bonds reported for this polysaccharide [30]. In particular, the base structure chosen for building this model corresponded to the carrisyn (acemannan) molecule (MF: C_66_H_100_NO_49_, MW: 1691.5 g/mol), as reported in the PubChem database under the compound CID number 72041 [45]. This molecule is a representative eight-sugar fragment of the acemannan typically found in the AV leaf mucilage, which is comprised of α-D-galacto mannan, α-D-galacto mannan acetate, and N-acetyl-D-glucosamine residues linked by α-1,4-glycosidic bonds. A two-dimensional representation used for the molecular modelling of acemannan is presented in Figure 2.

**Figure 3:**
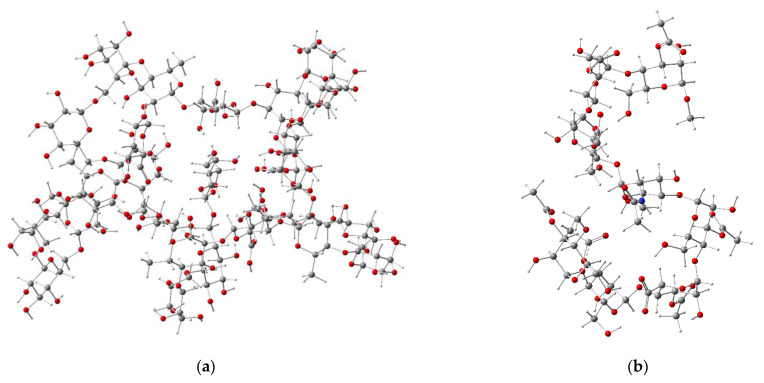
Stationary structures for the isolated molecules of OFI mucilage (**a**) and acemannan (**b**) minimized using the HF/6-31(d) level of approximation.

Figure 3 shows the 3D structures of the isolated molecules for the OFI mucilage and acemannan, optimized at the HF/6-31(d) approximation level. The stationary structure of the isolated OFI mucilage (Figure 3a) adopted a three-dimensional arrangement that resembles the shape of a “starfish”. Each of the five “extremities” in this structure corresponds to one of the Gal-β(1→6)Gal-β(1→6)Gal-β branched chains, which fold into the inner part, while the central chain forms a circle that is located in the outermost non-folded part. The folding on this structure is largely favored by the formation of intramolecular hydrogen bonds (HBs) between the carbonyl oxygen of the carboxyl group in L-rhamnopyranose central residues and hydroxyl oxygens (*>C=O···H–O*, 2.164 ≤ *d* ≤ 2.328 Å) or aliphatic hydrogens (*>C=O···H–C*, 2.278 ≤ *d* ≤ 2.377 Å) in D-galactopyranose branched-chain residues. A cavernous form, added to a high concentration of hydroxyl (OH) and ether (ROR’) groups in the folded part of this structure, together with the presence of some carbonyl groups (*C=O*), constitute a reticular system capable of coordinating a high number of water molecules, which explains the reservoir properties of the OFI mucilage.

On the other hand, the optimized isolated structure of acemannan acquired a wavy spatial orientation in the shape of the letter “s”, as shown in Figure 3b. The folding in this structure is governed by the formation of intramolecular HBs between the carbonyl oxygen and methyl hydrogens in acetyl groups *(>C=O···H–CH_2_*–, 2.402 ≤ *d* ≤ 2.843 Å) or with hydroxyl oxygens *(>C=O···H–O*, *d* = 1.931 Å), as well as a sequence of HBs between hydroxyl groups *(–(H)O···H–O*, 1.998 ≤ *d* ≤ 2.101 Å). The oxygens atoms of the carboxylate group in particular form a set of short HBs (1.884 ≤ *d* ≤ 1.970 Å) with hydroxyl hydrogens (*OC=O···H–O*) of the vicinal D-galacto mannan residues, whereas the amide hydrogen forms an HB with the hydroxyl group of another D-galacto mannan residue (*N–H···O(H)–*, *d* = 2.319 Å). As in the case of OFI mucilage, the optimized structure of acemannan exhibits a high capacity to retain water molecules.

### 3.2. Fourier-Transform Infrared (FTIR) Spectroscopy

**Figure 4:**
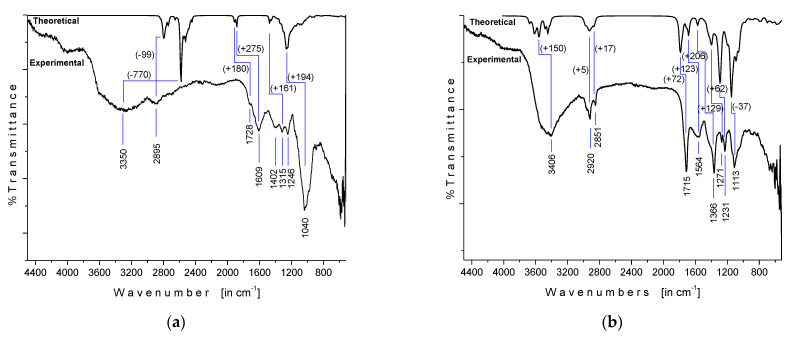
Experimental (in powder) and theoretical (in gas phase) IR spectra of OFI and AV mucilages: (**a**) FTIR spectrum of OFI mucilage versus the respective theoretical (unscaled) IR spectrum calculated at the HF/6-31(d)//PM6 level of approximation; (**b**) FTIR spectrum of AV mucilage versus the theoretical IR spectrum (scaled at factor 0.8953 [51]) of acemannan calculated at the HF/6-31-G(d) level of approximation. Theoretical vibrational shifting (∆υ in cm^–1^) are presented in parenthesis.

The experimental FTIR spectra of OFI and AV mucilages measured in powder, along with the respective theoretical gas-phase IR spectra, are shown in Figure 4. The OFI mucilage FTIR spectrum (Figure 4a) showed a broad band centered at 3350/cm that was attributed to a combined contribution of OH stretching modes from both alcohol (*R–OH*) and carboxylic acid (*–C(O)–OH*) moieties involved in the intramolecular OH bonding, while the absorption observed at 2895/cm was assigned to a sum of *C–H* stretches from both pyranose *CH* and glycoside *–OCH_2_* moieties [26,50]. The absorptions observed at 1728/cm (shoulder) and 1609/cm, as well as the band at 1402/cm, were assigned to the carbonyl (υ(*C=O*)) and *COO*− stretching modes, respectively, of the D-galactopyranosyl uronic acid residues [51]. The signals at 1315 and 1246/cm were attributed to characteristic stretching vibrations of the pyranose ring, whereas the intense band at 1040/cm was related to the polysaccharide backbone [52]. As shown in Figure 4a, the comparison between the experimental (in powder) FTIR spectrum and the theoretical (in gas-phase) IR spectra of OFI mucilage demonstrated positive vibrational shifting values (+∆υ) between 275 and 161 cm^–1^ for signals in the fingerprint region (2000 to 500 cm^–1^) and negative ∆υ values for absorptions located at higher frequencies. In particular, the semi-empirical PM6 method revealed a very poor estimation (∆υ = −770 cm^–1^) for the hydroxyl (*O–H*) harmonic frequencies, while the CH vibrations were the best approximate (∆ = −99 cm^–1^) in this macromolecule.

The FTIR spectrum of AV mucilage in powder (Figure 4b) showed a broad band centered at 3406/cm that was attributed to the combined presence of multiple hydroxyl groups. According to our ab initio calculations (HF/6-31(d)), the greatest contribution to this band can be attributed to the OH stretching modes belonging to the sequential chain of intramolecular (H)*O···H–O* bonds in acemannan, as explained above in Section 3.1. The absorptions observed at 2920 and 2851/cm were assigned to the aliphatic *C–H* stretching. The signals at 1715 and 1564/cm were attributed to *C=O* stretching and *COO–* asymmetric stretching, respectively, indicating the presence of carbonyl compounds (acemannan content). The bands at 1366, 1271, and 1231/cm were related to the asymmetric *CH_3_* scissoring, the bending (*C–O–C*) glycosidic symmetric stretching vibration, and the *C–O–C* stretching of acetyl groups, respectively, with the latter related to the bioactive acetylated polysaccharide acemannan. Finally, the absorption observed at 1113/cm was assigned to the *C–O* stretching [53,54]. As can be seen in Figure 4b, the comparison between the IR spectrum of the experimental AV mucilage and the theoretical spectrum of the isolated acemannan revealed a close correlation between them, which showed a high composition of acemannan in the dehydrated sample. Likewise, the theoretical spectrum (scaled at factor 0.8953, [50]) presented an overestimation of less than 200 cm^–1^ for all absorptions, which indicates a good vibrational prediction for this molecule using the approximation level HF/6-31(d).

### 3.3. Raman Spectroscopy

**Figure 5:**
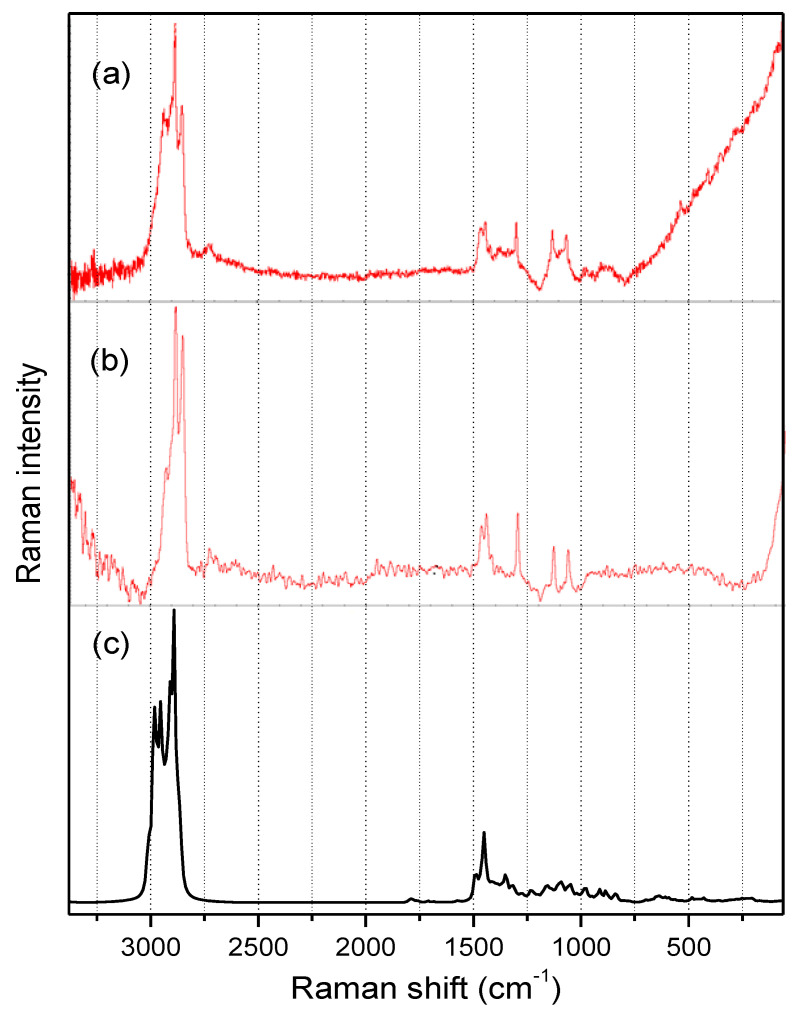
Raman spectra of powdered OFI (**a**) and AV (**b**) mucilage in the range of 3400 to 100/cm. The theoretical Raman spectrum for the isolated acemannan molecule (**c**), calculated at the HF/6-31G(d) approximation level and scaled at factor 0.8953 [50], is included for comparative purposes.

The Raman spectra of OFI and AV-powdered mucilages, along with the calculated (HF/6-31G(d)) Raman spectrum for isolated acemannan in the 100–3400/cm spectral region are shown in Figure 5a–c. Both OFI and AV mucilage’s spectra presented a similar well-resolved pattern with strong signals in the 2800–3000/cm range and medium-intensity signals in the 1000–1500/cm range. As is also noted in Figure 5, an excellent agreement was obtained when comparing the experimental Raman spectrum of the AV mucilage in powder (Figure 5b) and the Raman spectrum calculated for the acemannan molecule (Figure 5c), thus reaffirming the high content of acemannan in the sample of dry AV mucilage, and allowing the possibility of making more specific assignments of the signals in the Raman spectra. Consequently, the three narrow signals observed in both mucilage spectra in the υ(*C–H*) region around 2940, 2890, and 2850/cm were associated with the asymmetric mode of the hydrogen attached to the anomeric carbons, the asymmetric mode of the hydrogens of the pyranose ring, and the symmetric mode of the methyl hydrogens, respectively. The two sharp signals observed close to 1560 and 1540/cm in both mucilage’s Raman spectra were attributed to the symmetric deformations, ρ(*CH_2_*), in *–CH_2_OH* moieties, and ρ(*CH_3_*) in acetyl groups, respectively. The acute signal around 1300/cm was assigned to the υ(*C–C*) stretching mode in pyranose rings, whereas the two narrow signals around 1120 and 1050/cm were assigned to a complex motion involving the polysaccharide backbone and the υ(*C–O*) mode in the pyranose ring, respectively.

### 3.4. Microscopic Morphology

**Figure 6:**
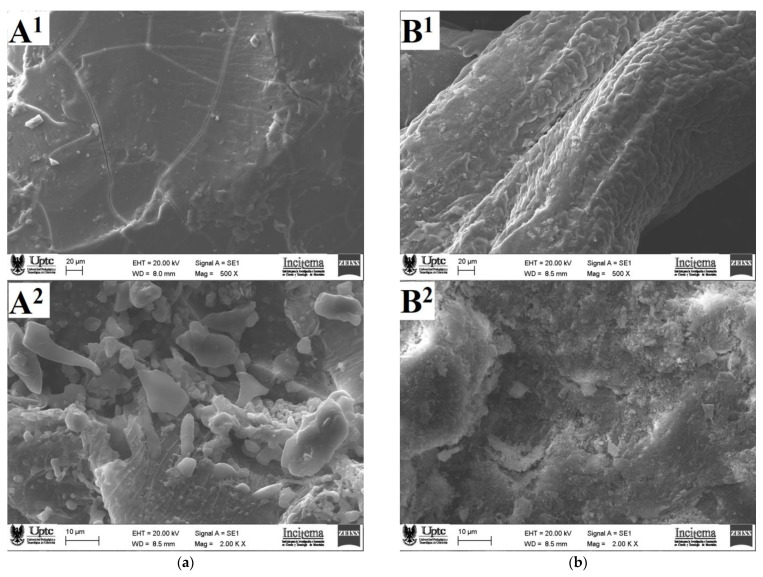
SEM micrograph images of the surface at 500× (1) and of the internal structure at 2000× (2) for mucilage of OFI (**a**) and AV (**b**) in powder.

Figure 6 shows SEM micrographs of the surface and the internal structures of the powered samples for OFI and AV mucilages obtained by a forced air convection drying process. The OFI mucilage sample observed with a magnification of 500× showed an irregular, compact, dense, and cracked surface that coincides with the morphological descriptions given previously for this kind of mucilages [27]. In contrast, the AV mucilage surface observed at 500× exhibited a rough morphology characterized by the presence of scales and pores.

Regarding the internal mucilage structures observed using a magnification of 2000×, the OFI sample showed the presence of small particles with diameters ranging between 2 and 10 μm that possibly correspond to protein aggregates adhered to the carbohydrate blocks, whereas the AV sample showed a heterogeneous slightly rough internal morphology with the presence of cavities. This last microstructure resembled the morphology recently described for mucilage samples extracted from the rhizomes of Taro (*Colocasia esculente*) by means of an ethanol precipitation [55]. The morphological differences observed between OFI and AV mucilages may be associated with the different conditions used during extraction processes, flocculation during ethanol precipitation, and sample dryness [56].

### 3.5. Thermal Properties

**Figure 7:**
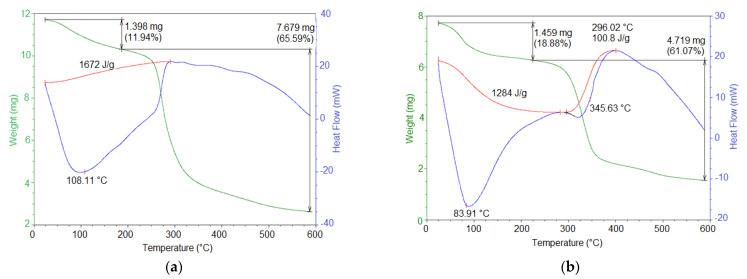
Representative TGA/DSC thermograms of OFI (**a**) and AV (**b**) mucilage samples in powder.

The thermal behavior of the OFI and AV mucilage powder samples was analyzed by DSC/TGA, resulting in the thermograms shown in Figure 7. The thermogram for OFI mucilage (Figure 7a) was characterized by two principal thermal events. First, an endothermic event occurred between 50 and 250 °C (peak 108.11 °C) with a related mass loss of 11.94%. This event was attributed to water loss followed by gelatinization. This moisture content was attributed to the hygroscopicity of the powdered mucilage. The second event corresponded to an exothermic process that occurred between 250 and 325 °C (peak 300 °C), with a mass loss of 65.59%. This event was attributed to the degradation of the polysaccharide structure and the subsequent decomposition/volatilization of material [18]. Similar thermal behavior has been previously reported for OFI mucilage samples [27].

On the other hand, the AV mucilage thermogram (Figure 7b) showed two endothermic events and one exothermic event. The first endothermic event occurred between 60 and 200 °C (peak 82 °C) and was attributed to the glass transition temperature. This event was accompanied by a related mass loss of 18.88% due to the loss of water (absorbed by hygroscopicity) and the decomposition of structural units. The second endothermic event was observed between 210 and 400 °C (peak 310 °C), which was assigned to the melting temperature. This event was accompanied by a related mass loss of 61.07% due to the thermal polymer degradation. The exothermic event occurred between 375 and 425 °C (peak 400 °C), which was attributed to a new crystallinity. This behavior was similar to that reported by Agha Mohammadi et al. for AV gel-dehydrated powder [57].

As described above, the AV mucilage showed lower thermal stability, as well as a lower melting and degradation temperature, compared to the OFI mucilage. This difference in thermal behavior between these two mucilages is related to the lower molecular weight and the hydrophilic nature of the functional groups of the AV mucilage biopolymer. These results revealed that OFI mucilage has a potential use in foods processed at high temperatures, while AV mucilage must be added to industrial processes at milder temperatures.

### 3.6. Dietary Fiber Content

The total dietary fiber content in the dry samples of OFI and AV mucilage was 73.9 g/100 g of powder and 37.0 g/100 g of powder, respectively. The greater dietary fiber content in OFI mucilage, resulted in its higher thermal stability and provides this biopolymer the advantage of being used in nutritional preparations with more potent prebiotic potential and better positive effects on the immune system [58]. Therefore, OFI mucilage should be considered as an optimal raw biomaterial to reduce the sugar and fat content in certain products.

## 4. Conclusions

Powder samples of mucilage from cladodes of *Opuntia ficus-indica* (OFI) and aloe vera (AV) leaves were extracted using precipitation in ethanol and dried by means of forced air convection techniques. Molecular characterization of these solid samples was performed using FTIR and Raman spectroscopy in combination with the execution of quantum-chemical calculations based on ab initio and semi-empirical theoretical methods. Computational modeling at the HF/6-31(d) approximation level, built using characteristic sugar residues and glycosidic connections found in this type of polysaccharide, led to representative three-dimensional stationary molecular structures for both mucilages. The isolated molecular models for OFI and AV mucilages adopted intricate structures, resembling the shape of a starfish and a wavy ribbon, respectively, both with an intrinsic capacity to coordinate a high number of water molecules. Such structures were rationalized based on different types of intramolecular hydrogen bonds, the formation of which was supported with the assignment of vibrational signals obtained by FTIR and Raman spectroscopies. The study of the microscopic morphology of the powdered mucilage samples using SEM revealed significant differences in the surface and internal structures of the OFI and AV samples. Such morphological changes were attributed to the different experimental conditions used during the extraction and drying processes of the mucilages. The OFI and AV mucilages obtained have a nutritional value due to the presence of total dietary fiber and polysaccharides, as revealed by enzymatic–gravimetric method and the infrared spectroscopy, respectively. According to the DSC/TGA and fiber content analysis, solid OFI mucilage had a greater thermal stability and dietary content, which makes this heteropolysaccharide a more convenient ingredient in medium–high temperature industrial preparations and with a greater nutritional benefit than powder AV mucilage. Nevertheless, it is worth mentioning that both OFI and AV powdered mucilages can offer high gelling and thickening capacities as well as various nutritional and health benefits. These benefits make them extremely desirable natural ingredients for the cosmetic, pharmaceutical, and food industries. Consequently, the powdered mucilage extracted from both OFI cladodes and AV leaf has the potential to replace starch in the formulation of beverages, soups, bakery products, and meat derivatives. Likewise, the incorporation of these mucilages rich in dietary fiber in food matrices will allow their consideration in products with the “healthy label”, which will benefit the health of the consumer.

## Data Availability

The data presented in this study are available on request from the corresponding author.
